# Menthol, a consumer product additive, adversely affects human embryonic stem cells via activation of TRPM8 and TRPA1 channels

**DOI:** 10.1093/stcltm/szae099

**Published:** 2025-03-26

**Authors:** Shabnam Etemadi, Prue Talbot

**Affiliations:** Department of Bioengineering, University of California, Riverside, CA 92521, United States; Department of Molecular, Cell and Systems Biology, University of California, Riverside, CA 92521, United States

**Keywords:** human embryonic stem cells, transient receptor potential channels, TRPA1 channel, TRPM8 channel, menthol, gastrulation, epiblast

## Abstract

Many electronic cigarettes (ECs) contain high concentrations of menthol. The effect of menthol on human embryos in pregnant women who vape is not well understood. Human embryonic stem cells (hESCs) (an epiblast model) were used to test the hypothesis that 6.4-640 nM and 19.2-192 µM menthol, which activates TRP (transient-receptor-potential) channels, alters calcium homeostasis in embryos and adversely affects processes that are critical to gastrulation. Micromolar concentrations of menthol inhibited mitochondrial reductase activity in hESCs, an effect that was blocked by TRPA1 and TRPM8 inhibitors. Pulsatile exposure to menthol elevated intracellular calcium primarily by activating TRPA1 channels at nanomolar concentrations and TRPM8 channels at µM concentrations. nM menthol significantly inhibited colony growth by activating TRPA1 channels, while both TRPA1 and TRPM8 were activated by µM menthol. Inhibition of colony growth was attributed to cell death induced by menthol activation of TRPA1 and TRPM8 channels. nM menthol altered colony phenotype by increasing the major/minor axis ratio via TRPA1 and TRPM8 channels. Both nM and µM menthol induced alterations in hESC colony motility, an effect that was blocked only by the TRPM8 inhibitor. The menthol-induced increase in intracellular calcium adversely influenced growth, death, and migration, processes that are critical in gastrulation. Menthol concentrations that reach embryos in women who vape are high enough to activate TRPA1 and TRPM8 channels and perturbed calcium homeostasis. Pregnant women who vape likely expose their embryos to menthol concentrations that are harmful. These data could help prevent birth defects or embryo/fetal death.

## Graphical Abstract

**Graphical Abstract:**
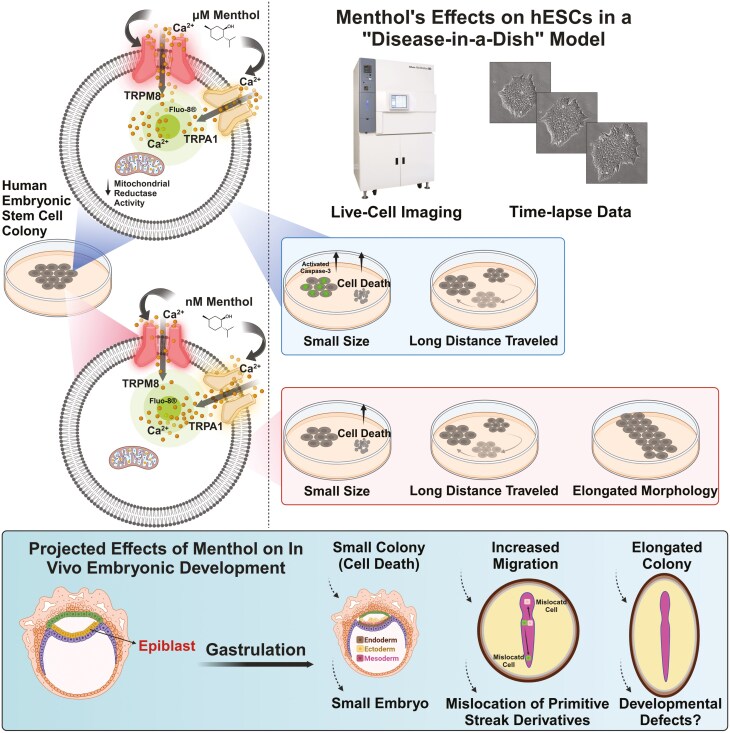

**Significance statement**

The influence of vaping on developing human embryos is poorly understood. Electronic cigarette aerosols have high concentrations of flavor chemicals, including menthol. We used human embryonic stem cells to model the epiblast stage of postimplantation development and tested the effects of low (nM) and high (µM) concentrations of menthol on processes critical to gastrulation. Our data show that menthol concentrations in the blood of women who vape during pregnancy are high enough to activate TRPA1 and TRPM8 channels in human embryonic stem cells and that activation decreases colony growth, increases cell death, and increases colony migration, effects that could lead to aberrant gastrulation.

## Introduction

Pregnant and reproductive-aged women are turning to electronic cigarettes (ECs) as a substitute for tobacco cigarettes to minimize the well-established developmental risks associated with smoking,^1-4^ such as preterm birth, low birth weight, and birth defects.^5-8^ However, the relationship between EC use and obstetric outcomes remains unclear. Concerns for both maternal and embryo/fetal health arise due to the potentially addictive, carcinogenic, and teratogenic nature of EC chemicals and metals^9-13^ and the reported health problems associated with vaping during pregnancy.^14^ Meta-analysis of data from the Pregnancy Risk Assessment Monitoring System (PRAMS) and the Population Assessment of Tobacco and Health (PATH) showed that pregnant women who vaped had increased odds of adverse perinatal outcomes (OR = 1.44).^15^ Subgroup analysis demonstrated that vaping during pregnancy was associated with 37% higher odds of pregnancy-related complications, and even higher odds (57%) of adverse neonatal outcomes. Individual analyses of specific perinatal outcomes identified significantly increased odds in low birthweight (OR = 1.64), small for gestational age (OR = 1.59), and preterm birth (OR = 1.39). In vitro data raised concerns about EC flavor chemicals adversely affecting embryonic development.^16^ Vaping mint or menthol-flavored ECs before and during pregnancy was associated with much higher fetal death (OR = 3.27) than vaping candied-flavored ECs (OR = 1.27).^17^ The reported linkage between EC menthol and increased fetal death may also be important for pregnant women who use traditional cigarettes as ~90% of tobacco products contain some menthol.^18^ Menthol concentrations in ECs are often much higher than those in other consumer products,^19-21^ demonstrating the need for a better understanding of the effects of menthol on prenatal development.

Menthol activates transient receptor potential (TRP) channels, specifically TRPM8 and TRPA1, which are permeable to Ca^2+^ ions.^22-24^ 1-100 µM concentrations of menthol activate TRPA1 channels in mouse cultured cells, while higher concentrations (>1 mM) can block TRPA1 channels.^25^ TRP channels are present throughout the reproductive tissues^26^ and in non-human embryos and placentas.^27-30^ However, little is known about TRP channels in human embryos and how they respond to menthol at concentrations that reach an embryo in pregnant women who vape. Because calcium ions play a critical role in signaling pathways in stem cells and differentiation,^31^ menthol activation of TRP channels in human embryos could alter precisely regulated steps in prenatal development leading to adverse birth outcomes.

The purpose of this study was to determine if menthol harms human embryos by activating TRPA1 and TRPM8 channels using concentrations of menthol that would be found in the blood of pregnant women who vape and human embryonic stem cells (hESCs), which are equivalent to epiblast cells in young post-implantation embryos.^32,33^ During weeks 3-4 of development, epiblast cells undergo gastrulation to produce ectoderm, endoderm, and mesoderm.^34^ The effects of menthol on TRPA1 and TRPM8 channels were studied using endpoints that are important during gastrulation. Errors in gastrulation can lead to adverse pregnancy outcomes, including miscarriage and fetal defects.^35^

## Materials and methods

All reagents, media, antibodies, consumables, software, and equipment are provided in Supplementary Table S1.

### Single-cell RNA-sequencing data analysis

A published human single-cell RNA-sequencing (scRNA-seq) dataset^36^ containing the transcriptomes of oocytes, early stages of embryonic development, and hESCs (passage 0 and passage 10) was mined to determine which TRP channels are present in each developmental stage. Count data (reads/kilobase of transcript/million mapped reads, RPKM) were processed using the Python package Scanpy.^37^ Cells were excluded if they exhibited fewer than 1000 detected transcripts. Genes with limited read counts (0-10 per at least 10 cells) were excluded from the analysis. Count data were log-normalized, and highly variable transcripts were chosen based on analytic Pearson residuals.^38^ To visualize the expression of TRP genes per cluster, heatmaps, and dot plots were used.

### hESC cell culture

H9 hESCs (female) obtained from WiCell were cultured in mTeSR plus medium on hESC-qualified Matrigel-coated wells as described previously^39-41^ and in the online supplement (Supplementary Text S1).

### Immunocytochemistry labeling and analysis

H9 hESCs were seeded in 8-well chamber slides. At 80% confluency, cells were fixed in 4% paraformaldehyde for 30 minutes at room temperature and washed extensively with PBS. Samples were permeabilized with 0.1% Triton X-100 and non-specific binding was blocked using 10% donkey serum. Further steps in labeling and analysis are described in the online supplement (Supplementary Text S2).

### Development and use of exposure model software

Exposure model software, written in the MATLAB R2022b programming environment, was developed to estimate blood concentrations of menthol in pregnant women who vape. The MATLAB source code and a stand-alone executable version are available upon request. The stand-alone executable requires the 64-bit version of MATLAB Runtime R2022b available at https://www.mathworks.com/products/compiler/matlab-runtime.html.

Menthol retention in the lungs was assumed to be 96% based on human participants who vaped JUUL “Menthol” ECs.^42^ The model was run using different concentrations of menthol that were determined in EC liquids.^19,20^ The puff number was varied between 1 and 27.

To determine the mass of menthol retained (mg), the concentration of menthol in EC-liquid (mg/mL) was multiplied by the average mass of the liquid consumed (mg) during vaping and then divided by the density of the EC-liquid (mg/mL). This product was multiplied by percent retention (Equation 1).


$$\begin{array}{llll} \; & Mass\;retained\;\left(mg\right)\; & =\;\%\;Retention\times \frac{Mass\;\;liquid\;\;consumed\;\;\;\left(mg\right)}{Density\;of\;\;EC\;liquid\;\;\left(\frac{mg}{mL}\right)}\; & \times Menthol\;\;in\;EC\;\;Liquid\;\left(mg/mL\right) \end{array}$$ (1)

Menthol concentration in maternal blood was estimated by dividing the mass retained (Equation 1) by plasma volume at week 2 of pregnancy (~ 2650 mL)^43^ (Equation 2). It was assumed that 50% of the menthol in the lung transferred to the mother’s blood.


$$\begin{array}{lll} \; & Menthol\;concentration\;in\;maternal\;blood\;\left(mg/mL\right)\; & =\frac{\;\%\;\;\;Transfer\;\;\;to\;blood\times mass\;retained\;\left(mg\right)}{Plasma\;volume\;\;\left(mL\right)} \end{array}$$ (2)

Menthol concentration in maternal blood produced by a single puff was estimated by dividing the menthol concentration retained in maternal blood (Equation 2) during vaping by the total number of puffs taken (Equation 3).


$$\begin{array}{lll} \; & Menthol\;\;concentration\;in\;\;single\;puf\;f\;\left(mg/mL\right)\; & =\frac{Concentration\;\;retained\;in\;\;\;maternal\;blood\;\;\;\left(mg/mL\right)}{Total\;number\;of\;puf\;fs} \end{array}$$ (3)

### Mitochondrial reductase activity measured using the MTT assay

The MTT assay was used to evaluate the effect of menthol (*L*(−)-menthol) on mitochondrial reductases in hESCs. Details of the assay are published^16^ and are in the online supplement (Supplementary Text S3).

### Calcium influx assay

Menthol-induced calcium influx in hESCs was measured using the Fluo-8 No Wash Calcium Assay Kit according to the manufacturer’s instructions. Cells (2 × 10^4^ cells/well) were grown to 70%-80% confluency in 96-well clear bottom black plates and then loaded with 1 µM Fluo-8 in HHBS buffer (Hanks’ Buffer with 20 mM HEPES C_8_H_18_N_2_O_4_S) for 20 minutes at 37°C. Baseline fluorescence was recorded for a duration of 20 seconds, followed by a 4-minute measurement after the addition of menthol at 37°C using a Synergy HTX Microplate Reader. nM and µM menthol concentrations were prepared as serial dilutions from 6.4 mM stock solutions in dimethyl sulfoxide (DMSO). In some experiments, cells were pre-incubated with the TRPA1 inhibitor (AM0902) and/or TRPM8 inhibitor (TC-I 2014) for 20 minutes prior to Fluo-8 dye excitation in the presence of inhibitors.

To confirm TRP channel inhibitor results, hESCs were pre-incubated with a TRPA1 antibody no. ACC-037, TRPM8 antibody^44^ no. ACC-049, or rabbit IgG isotype control for 20-30 minutes before the addition of Fluo-8 dye solution containing the antibodies. Stock solutions of the antibodies were diluted to the desired final concentrations in HHBS buffer. Intracellular Ca^2+^ changes were analyzed using MATLAB R2022b and plotted using GraphPad Prism.

### Live cell imaging assay and analysis

hESCs (1 × 10^5^ cells/well) were attached for 48-72 hours at 37°C in 12-well plates. The cells were then exposed to nM or µM menthol for 48 hours in a Nikon BioStation CT incubator with a microscope and camera that collected time-lapse images while maintaining proper humidity, CO_2_, and temperature.^45,46^ Phase-contrast images were captured every 4 hours using 2 × 2 tiling at 10X magnification for 48 or 72 hours. Nine colonies that remained in the field throughout the entire incubation period were analyzed for each control and experimental condition. In some experiments, cells were pre-incubated with the TRPA1 inhibitor (AM0902) and/or TRPM8 inhibitor (TC-I 2014) for 20 minutes prior to menthol treatment in the presence of inhibitors. BioStation images were combined into videos using CL Quant software^47^ and analyzed using StemCellQC.^48^ Graphs depicting dynamic and morphological features were generated using GraphPad Prism. Time-lapse videos were used to analyze growth (colony area), cell proliferation, cell death (brightness/total area ratio), colony morphology (major/minor axis ratio), and motility (total displacement and total distance traveled).^48,49^

### Cell proliferation assay

Cell proliferation was assessed with an antibody to Ki-67, a protein that labels nuclei with patterns characteristic of each stage in the cell cycle.^50^ hESCs (5 × 10^4^ cells/well) were cultured in 8-well chamber slides until they reached 70%-80% confluency, then incubated for 48 or 72 hours with nM or µM menthol made from a 6.4 mM stock in DMSO. Cells were then labeled with a Ki-67 antibody followed by an Alexa fluor-488 secondary antibody and imaged with a Nikon Eclipse Ti inverted microscope equipped with a high-resolution Andor Zyla VSC-04941 camera. Nuclear patterns of Ki-67 immunofluorescence staining were used to classify hESCs into different stages of the cell cycle. Three independent experiments were performed, and 100 cells were counted in each group in each experiment using ImageJ.

To validate the Ki-67 data, the mitotic index was measured in control and menthol-treated groups. For each condition, 100 cells were evaluated in 3 independent experiments, for a total of 300 cells/group. Mitotic cells in metaphase and anaphase were identified by their characteristic nuclear morphology using DAPI staining, and counts were done using the Cell Counter plugin in ImageJ. Prior to mitotic index analysis, dead cells were excluded from the counts by staining for activated caspase-3 (Asp175), an apoptosis marker. The mitotic index was calculated as the percentage of live cells in metaphase and anaphase relative to the total number of viable cells.

### Cell death assay

To complement data obtained using brightness/total area ratio, cell death was also monitored using an antibody to activated caspase-3, a cysteine-aspartic acid protease that executes cell death.^51^ hESCs (5 × 10^4^ cells/well) were grown in 8-well chamber slides for 48 hours, followed by treatment with menthol (19.2 µM, 64 µM, and 192 µM) or 1 µM hydrogen peroxide (H_2_O_2_) for 48 hours. In some experiments, cells were pre-incubated with the TRPA1 inhibitor (AM0902) or TRPM8 inhibitor (TC-I 2014) for 20 minutes prior to menthol treatment in the presence of inhibitors. Cells were then fixed in 4% paraformaldehyde and labeled with a rabbit polyclonal antibody to activated caspase-3 (1:200) and an Alexa fluor-488 secondary antibody. Immunofluorescent cells were imaged using a Nikon Eclipse Ti inverted microscope equipped with a high-resolution Andor Zyla VSC-04941 camera.

A CL Quant protocol was applied to remove background noise and measure fluorescence intensity in 100 cells in each group for each experiment. Three independent experiments were performed. The percentage of positively stained cells was also quantified using the Cell Counter plugin in ImageJ with *n* = 100 cells/group/experiment. These measurements were imported into GraphPad Prism for graphing and analysis.

### Data analysis and statistics

All experiments were performed with 3 different passages of H9 hESCs. Statistical data are presented as the mean ± the standard error of the mean (SEM) using GraphPad Prism software. In the MTT, calcium influx, proliferation, and cell death assays, statistical analyses were performed in GraphPad Prism using a one-way ANOVA followed by Dunnett’s or Tukey’s posthoc test. Live cell imaging data were analyzed using a two-way ANOVA with Dunnett’s posthoc test, in which the factors were time and treatment. If data did not meet the assumptions of ANOVA (normality of distribution and homogeneity of variances), they were subjected to a log(*y*) transformation. Means were considered significantly different for *P* < .05.

## Results

### TRP channels in early stages of human development

**Figure 1.**
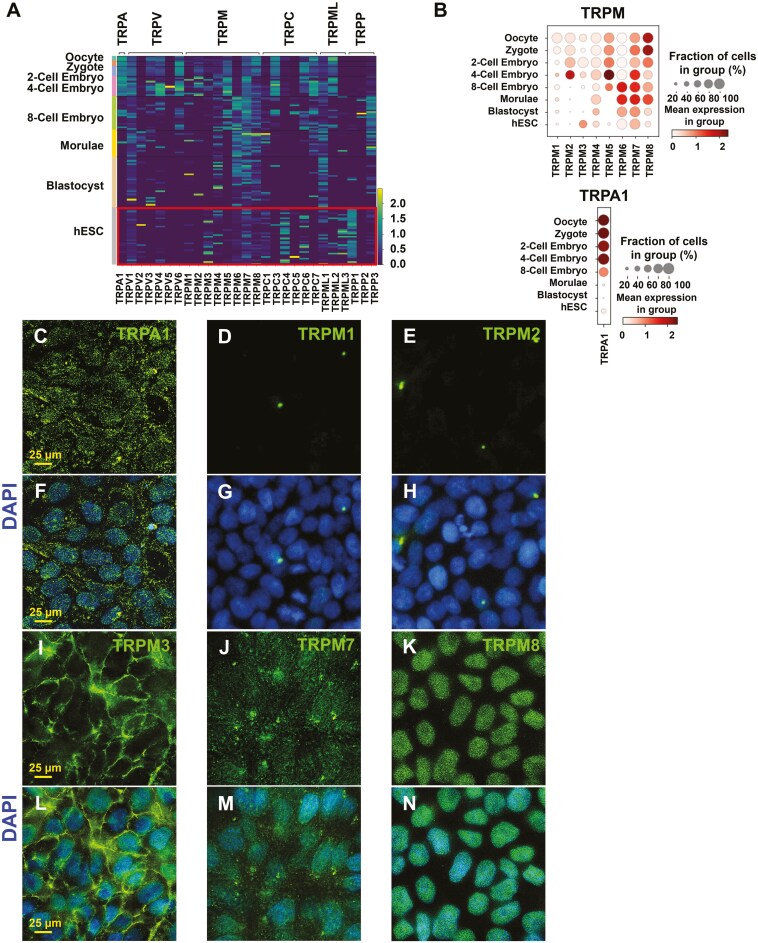
TRP channels in early stages of human development and H9 hESCs. (A) Heatmap showing 27 differentially expressed TRP genes in 124 cells mined from a scRNA-seq dataset based on RPKM values. Log2 RPKM values are plotted on a high-to-low scale (yellow-green-blue) for eight stages of development. (B) Dot plots showing the expression of TRPM and TRPA1 genes in 8 developmental stages. Average gene expression levels and the percentage of cells that express each gene are presented with differential color intensities and circle sizes, respectively. (C-N) Representative immunofluorescence images of H9 hESCs labeled with specific antibodies (green) to TRPA1, TRPM1, TRPM2, TRPM3, TRPM7, and TRPM8 channels. (F-H) and (L-N) TRP labeling merged with DAPI (blue) to show nuclei. Scale bar indicates 25 µm.

A human oocyte/embryo scRNA-seq dataset^36^ was used to assess mRNA levels of TRP channels in human oocytes, zygotes, 2-cell embryos, 4-cell embryos, 8-cell embryos, morulae, blastocysts, and hESCs (Figure 1A, B). Transcripts for 27 TRP channels varied in abundance with each developmental stage. In the hESC group, mRNA transcripts for TRPM1, TRPM2, and TRPM5 were sparse or absent. mRNA levels of TRPM3 and TRPM7 were elevated in most hESCs (Figure 1B), while TRPM4, TRPM6, TRPM8, and TRPA1 were present in relatively low abundance (Figure 1B).

Immunocytochemistry was performed to confirm the presence of TRP proteins in hESCs and determine their location (Figure 1C-N). In agreement with the scRNA-seq data, TRPA1, TRPM3, TRPM7, and TRPM8 proteins were present in hESCs, while TRPM1 and TRPM2 were not detected. The relative abundance of TRPA1 and TRPM8 was higher in the protein data than in the scRNA-seq analysis. The location of TRP channels varied with TRPA1 (Figure 1C, F) and TRPM7 (Figure 1J, M) being homogenously distributed on the cell, TRPM3 (Figure 1I, L) being restricted to boundaries between cells, and TRPM8 (Figure 1K, N) being concentrated in the region above the nuclei.

### Estimation of menthol concentrations in maternal blood

The concentration of menthol in the blood of a pregnant woman who is vaping was estimated to determine concentrations for use in subsequent experiments (Supplementary Figure S1A). Estimations were based on scenarios in which the concentration of menthol in EC-fluids and the number of puffs were varied. At week 2 of pregnancy, for a woman vaping 1 puff of an EC with a menthol concentration of 3 mg/mL and 50% transfer from lungs to blood, the estimated concentration of menthol in maternal blood was 11.62 nM. Maternal blood concentration increased to ~2825 nM when the mother took 27 puffs of an EC containing 27 mg/mL of menthol. The half-life of menthol is <1 hour.^52^ If vaping occurred every hour, the blood concentration of menthol would gradually increase over time (Supplementary Figure S1B). Subsequent experiments were conducted using nanomolar (nM) and micromolar (µM) menthol that bracketed concentrations estimated to be in maternal blood during vaping.

### Micromolar menthol inhibited mitochondrial reductase activity by activating TRPM8 and TRPA1 channels

**Figure 2.**
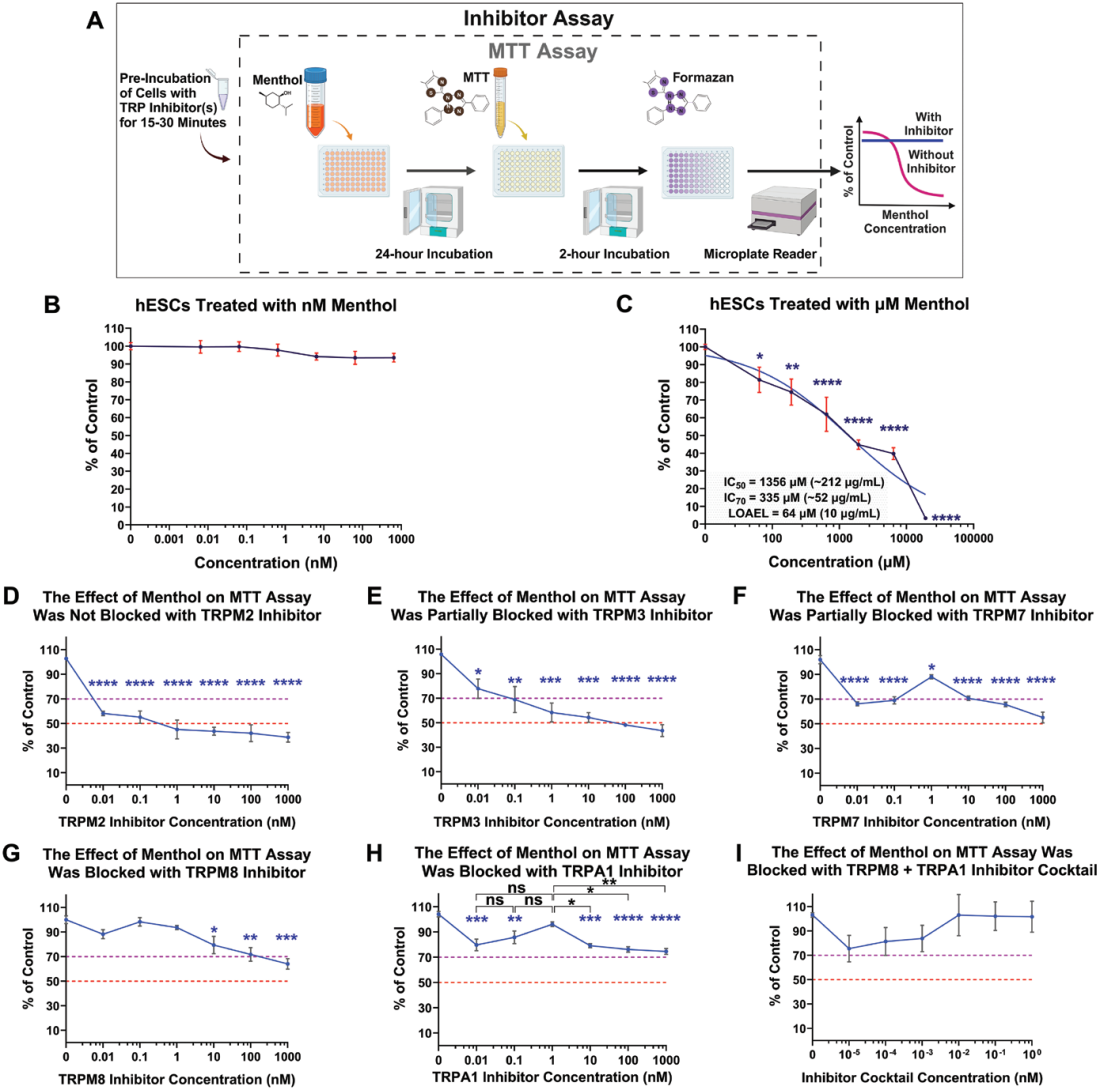
Menthol inhibited mitochondrial reductases in hESCs by activating TRP channels. (A) Schematic of MTT and inhibitor assay used to determine activation of TRP channels by menthol in hESCs. (B) nM menthol did not affect mitochondrial reductases. DMSO vehicle control had no significant effect on the MTT assay (data not shown in the plot). (C) µM menthol inhibited mitochondrial reductases. The blue curve is a line fitted to the data using nonlinear regression. The IC_50_ concentration was determined with GraphPad Prism using nonlinear regression (dose-response-inhibition: [Inhibitor] vs response—variable slope (four parameters)). The IC_70_ was derived from the IC_50_ using the equation $\;IC_{70}={\left(\frac{70}{100-70}\right)}^{\frac{1}{Hill\;\;\;Slope}}\times IC_{50}$ provided by GraphPad Prism. The LOAEL concentration is the lowest concentration of menthol that produced a significant effect vs untreated control. (D-I) Concentration-response curves for µM menthol when tested in the presence of inhibitors for TRPM2 (D), TRPM3 (E), TRPM7 (F), TRPM8 (G), TRPA1 (H), and a cocktail of TRPM8 and TRPA1 (I). Red and purple dashed lines show the MTT IC_50_ and MTT IC_70_ concentrations for menthol, respectively. Data were plotted as a percentage of the untreated control and are means of 3 independent experiments ± SEM (standard error of the mean) for each concentration. A one-way ANOVA was performed with Dunnett’s posthoc comparisons to the mean of the untreated controls, except in (H), in which Tukey’s posthoc test was used to compare adjacent concentrations. *P < .05, **P < .01, ***P < .001, ****P < .0001. n.s. = not significant.

The effect of menthol on mitochondrial reductase activity was evaluated with the MTT assay using the experimental design in Figure 2A.^53^ While nM menthol did not affect the MTT assay (Figure 2B), µM menthol inhibited mitochondrial reductases in a concentration dependent manner (Figure 2C). hESCs were pre-incubated with TRP inhibitors and then exposed to the MTT IC_50_ concentration of menthol in the presence of inhibitors. Figure 2D-I shows the IC_50_ value (red dotted line), the IC_70_ value (purple dotted line), and the actual response (solid blue line) of cells exposed to the IC_50_ concentration of menthol in the presence of inhibitors to TRPM2 (Figure 2D), TRPM3 (Figure 2E), TRPM7 (Figure 2F), TRPM8 (Figure 2G), and TRPA1 (Figure 2H). Inhibition of mitochondrial reductases by menthol was not blocked by the TRPM2 inhibitor (tatM2NX) and was only partially blocked by the TRPM3 inhibitor (mefenamic acid) or the TRPM7 inhibitor (CCT128930) (Figure 2D-F). The inhibitors of TRPM8 (TC-I 2014) and TRPA1 (AM0902) were the most effective and significantly blocked the effect of menthol when used as a cocktail (Figure 2G-I). Since inhibitors of TRPM8 and TRPA1 channels blocked the effect of menthol in the MTT assay, the remaining experiments were done using nM and µM menthol in conjunction with these inhibitors.

### Menthol-induced calcium influx in hESCs through activation of the TRPM8 and TRPA1 channels

**Figure 3.**
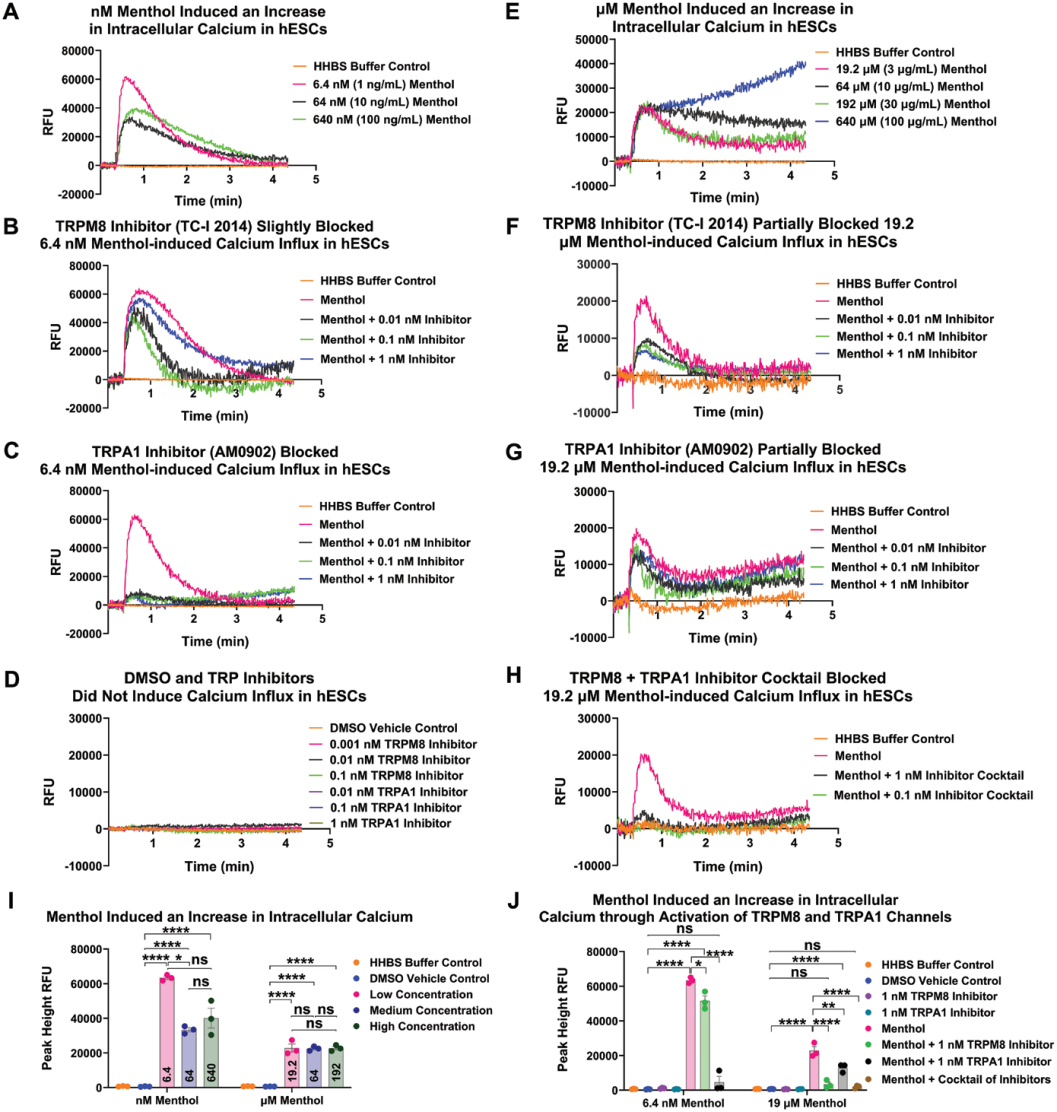
Menthol-induced calcium influx by activation of TRPM8 and TRPA1 channels. (A) Representative graph showing menthol induced an increase in intracellular calcium at nM concentrations (6.4-640 nM). (B) TRPM8 inhibitor (TC-I 2014) blocked (~19%) calcium influx induced by 6.4 nM menthol. (C) TRPA1 inhibitor (AM0902) significantly (~93%) blocked the influx of calcium induced by 6.4 nM menthol. (D) DMSO vehicle control and various concentrations of TRPM8 inhibitor (TC-I 2014) and TRPA1 inhibitor (AM0902) did not induce an increase in intracellular calcium in hESCs. (E) Menthol induced an increase in intracellular calcium at µM concentrations (19.2-640 µM). (F) TRPM8 inhibitor (TC-I 2014) partially blocked (~85%) the influx of calcium induced by 19.2 µM menthol. (G) TRPA1 inhibitor (AM0902) partially blocked (~43%) the influx of calcium induced by 19.2 µM menthol; however, calcium levels increased in cells at later times, even in the presence of the inhibitor. (H) A cocktail of TRPM8 inhibitor (TC-I 2014) and TRPA1 inhibitor (AM0902) significantly blocked (~90%) the influx of calcium induced by 19.2 µM menthol. Each experiment was done 3 times, and each graph shows a representative experiment. The RFU for each graph is baseline subtracted. (I) Mean peak heights for hESCs treated with nM or µM menthol. (J) Effect of TRPM8 (TC-I 2014) and TRPA1 (AM0902) inhibitors on mean peak heights. All one-way ANOVAs were done on transformed (log(*y*)) data. *P < .05, **P < .01, ***P < .001, ****P < 0.0001 determined by Tukey’s posthoc test. n.s. = not significant.

nM menthol (6.4-640 nM) caused an immediate robust increase in [Ca^2+^]_*i*_ that peaked at about 1 minute and then declined to baseline by 4 minutes (Figure 3A). The [Ca^2+^]_*i*_ peak induced by 6.4 nM menthol was decreased by about 19% when the TRPM8 inhibitor (TC-I 2014) was included in the culture medium (Figure 3B). In contrast, the TRPA1 inhibitor (AM0902) reduced the peak by 93% (Figure 3C). The DMSO vehicle control and serial dilutions of the TC-I 2014 and AM0902 inhibitors did not affect [Ca^2+^]_*i*_ in hESCs (Figure 3D). Thus, nM concentrations of menthol caused significant calcium influx by activation of the TRPA1 channels, with a minor contribution by TRPM8. µM menthol (19.2 µM-640 µM) elevated intracellular calcium, although peaks were lower than those with nM concentrations (Figure 3E). Peaks were maximal by about 1 minute, after which they declined, although not to the baseline. At 640 µM, relative fluorescence units (RFUs) continued to elevate over 4 minutes of recording. When experiments were repeated in the presence of the TRPM8 channel inhibitor (TC-I 2014), the [Ca2^+^]_*i*_ peak triggered by 19.2 µM menthol was reduced by about 85% (Figure 3F). The TRPA1 inhibitor (AM0902) also decreased calcium influx (~43%) but was less effective than the TRPM8 blocker (Figure 3G). When hESCs were preloaded with both channel inhibitors, calcium influx was reduced to the level of the untreated control (Figure 3H). These data show that µM menthol elicits [Ca^2+^]_*i*_ in hESCs via both the TRPM8 and TRPA1 channels with the TRPM8 being more responsive.

The calcium peak height was significantly elevated in hESCs exposed to various menthol concentrations, with the effect being strongest in 6.4 nM menthol (Figure 3I). While both nM and µM concentrations of menthol increased calcium peaks, these increases were significantly inhibited by the TRPA1 inhibitor (AM0902) in response to nM menthol and by the TRPM8 inhibitor (TC-I 2014) or a cocktail of inhibitors in response to µM menthol (Figure 3J).

To confirm the TRP channel inhibitor data, experiments were done with function-blocking antibodies to TRPA1 (ACC-037) and TRPM8 (ACC-049) channels. The antibodies alone (3 µM of ACC-037, ACC-049, and control rabbit IgG) showed no effect on intracellular calcium levels in hESCs (Supplementary Figure S2A). When hESCs were exposed to 6.4 nM or 19.2 µM menthol, the antibody to the TRPA1 channel (ACC-037) reduced calcium influx by ~95% and ~56% respectively, similar to data obtained with the TRPA1 channel blocker (Figure 3J). The antibody to TRPM8 channels (ACC-049) significantly inhibited (~92%) calcium influx by 19.2 µM menthol but had a lesser effect (~38%) on the calcium response induced by 6.4 nM menthol (Supplementary Figure S2B). These data show that nM menthol induced Ca^2+^ influx preferentially through TRPA1 channels, while µM menthol triggered an influx of Ca^2+^ primarily in a TRPM8-dependent manner, consistent with findings using TRP inhibitors (Figure 3). The same concentration (3 µM) of the control IgG antibody had no effect on the activation of TRPA1 and TRPM8 channels by menthol (Supplementary Figure S2B).

### Menthol inhibited hESC colony growth through activation of TRPM8 and TRPA1 channels

**Figure 4.**
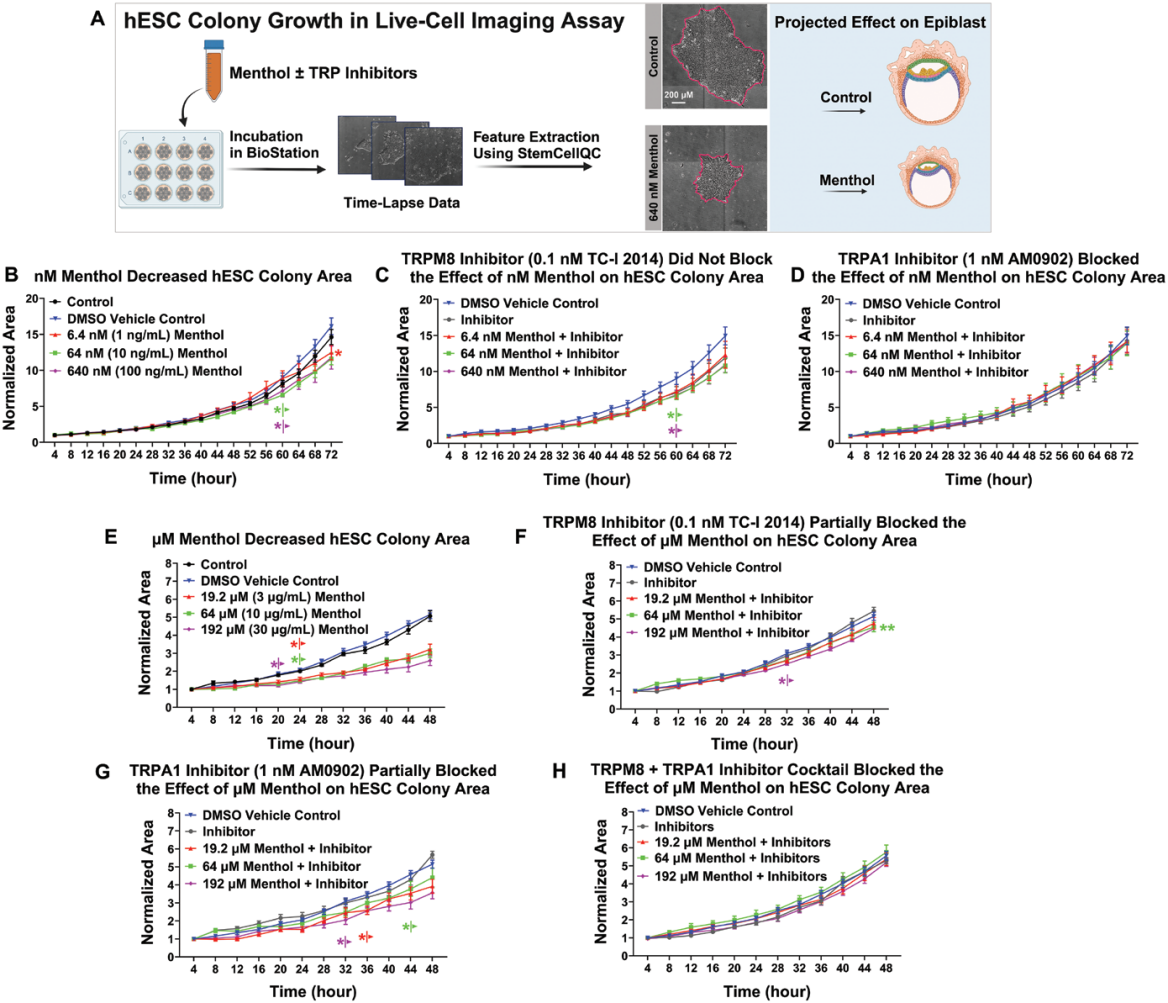
Menthol inhibited hESC colony growth through activation of TRPM8 and TRPA1 channels. (A) Schematic representation of live-cell imaging assay used for hESC colony growth. Time-lapse data were collected in a BioStation CT for 48 or 72 hours, and normalized area was extracted using StemCellQC software. Changes in hESC colony growth were projected into possible changes in epiblast growth. In all graphs, data were normalized to the first time point. (B) Area for control and nM menthol-treated colonies. nM menthol (6.4-640 nM) decreased hESC colony area significantly. (C) TRPM8 inhibitor (0.1 nM TC-I 2014) did not block the effect of nM menthol on hESC colony area. (D) TRPA1 inhibitor (1 nM AM0902) blocked the effect of nM menthol on hESC colony area. (E) µM menthol decreased hESC colony area significantly for all menthol concentrations (19.2-192 µM). (F) TRPM8 inhibitor (0.1 nM TC-I 2014) partially blocked the effect of µM menthol on hESC colony area. (G) TRPA1 inhibitor (1 nM AM0902) partially blocked the effect of µM menthol on hESC colony area. (H) A cocktail of TRPM8 (0.1 nM) and TRPA1 (1 nM) inhibitors blocked the effect of µM menthol on hESC colony area. Data are plotted as means of 3 independent experiments ± SEM for each concentration. Data in (B) and (C) were transformed (log(*y*)) to satisfy the assumptions of ANOVA. Arrows indicate first values that differed significantly from the DMSO vehicle control by 2-way ANOVA with Dunnett’s posthoc test. *P < .05, **P < .01.

To determine if menthol activation of TRP channels affected hESC colony growth, time-lapse data were collected every 4 hours, and area (total number of pixels inside a segmented colony) was extracted using StemCellQC software and normalized to the first time point^48^ (Figure 4A). The untreated and DMSO controls grew well during 72 hours of in-vitro culture and did not differ significantly from each other. In contrast, colony area decreased significantly in the 6.4, 64, and 640 nM menthol groups compared to the DMSO control (Figure 4B). This reduction in growth was not prevented in the 64 and 640 nM menthol treatments by the TRPM8 channel blocker (TC-I 2014) (Figure 4C) but was significantly inhibited by the TRPA1 inhibitor (AM0902) at all nM concentrations of menthol (Figure 4D).

The 3 µM concentrations of menthol significantly inhibited colony growth (area) during 48 hours of in-vitro incubation (Figure 4E). The effect on growth was partially blocked by the TRPM8 inhibitor (Figure 4F) and TRPA1 inhibitor (Figure 4G) when tested independently and completely blocked when both inhibitors were combined (Figure 4H), supporting the idea that both TRPM8 and TRPA1 channels contribute to µM menthol-induced decreases in hESC colony growth.

### Menthol did not cause a biologically significant effect on cell proliferation

**Figure 5.**
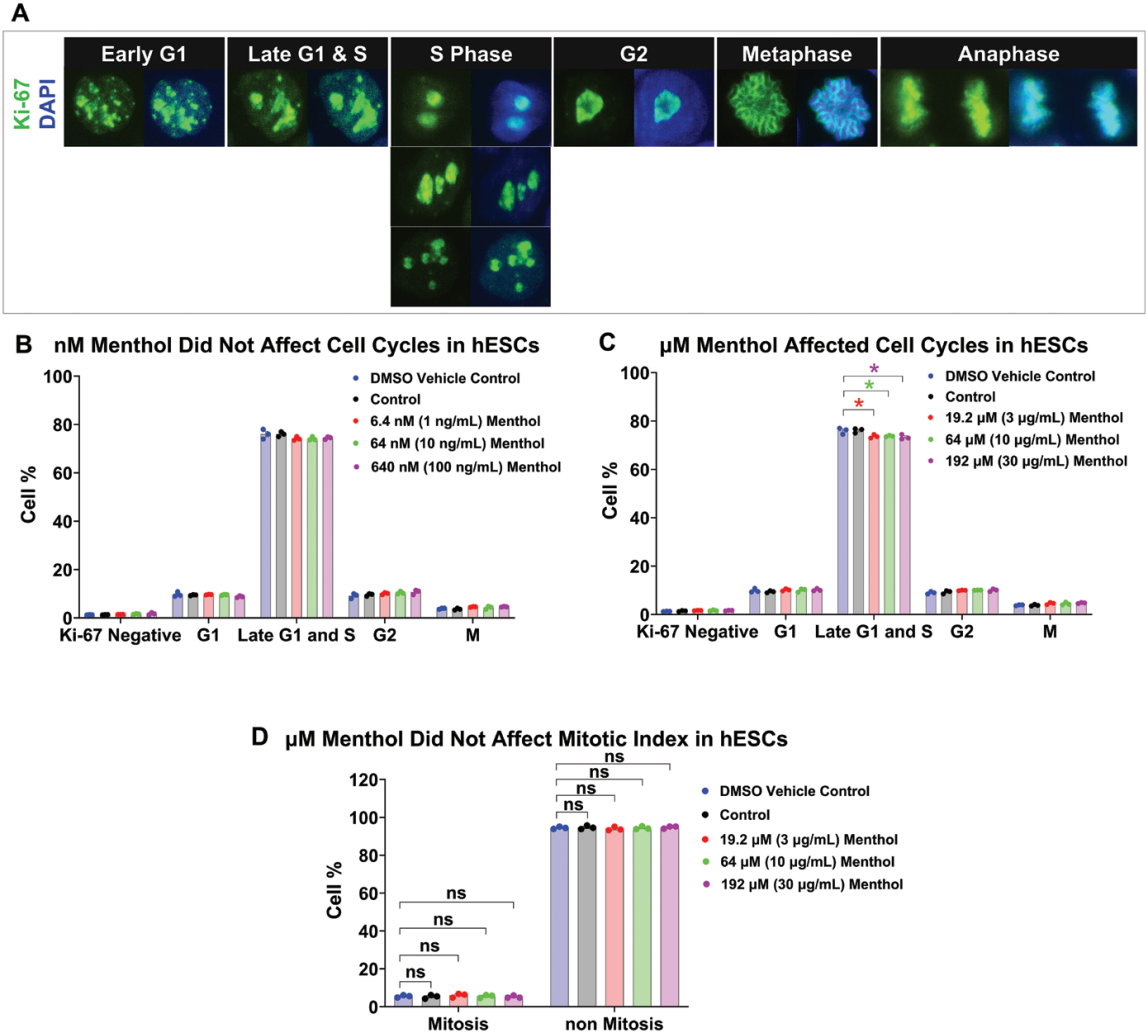
Menthol did not inhibit stem cell proliferation. (A) Nuclear detection of Ki-67 protein in hESCs. Ki-67 nuclear staining patterns (*n* = 100 cells/group/experiment) were used to identify different stages of the hESC cell cycle. (B) Distribution of hESCs in different cell cycle stages following 72 hours of exposure to nM menthol which caused no significant changes in the number of cells in different stages. (C) Distribution of hESCs in different cell cycle stages following 48 hours of exposure to µM menthol which significantly decreased the number of cells in the late G1 & S stages. Decreases in G1 & S were small and not likely biologically significant. (D) Mitotic index was not significantly changed following 48 hours of exposure to µM menthol (*n* = 100 cells/group/experiment). Mitosis = metaphase and anaphase stages; non-mitosis = all other stages except metaphase and anaphase. A one-way ANOVA was performed with Dunnett’s post hoc comparisons to the DMSO vehicle control. *P < .05.

To determine if menthol inhibited cell growth by inhibiting cell proliferation, the subnuclear organization of Ki-67 was examined in control and treated hESCs (Figure 5A). The pattern of Ki-67 immunostaining changes with each stage of the cell cycle^50^ and was used to determine the number of cells/stage. All cells exhibited positive staining with the human Ki-67 antibody, indicating the absence of quiescent (G0) cells (Figure 5A). The percentage of cells varied in each phase of the cell cycle with late G1/S having the highest percentage (72.1 ± 0.6%). However, within each phase, the percentages of cells in the control vs the treated groups were very similar (eg, in late G1/S, all groups had about 73%-75% of the cells in the population) (Figure 5B, C). While there was a statistically significant difference in the control vs µM treated cells in the late G1/S phase, the differences between the means in these groups were very small and probably not biologically significant (eg, in the group treated with µM menthol, the DMSO control = 76% and the 192 µM = 73.4%).

To confirm the results with Ki-67, the mitotic index was scored for cells exposed to different concentrations of menthol using samples that were labeled with an antibody to activated caspase-3 so that dead cells could be eliminated from the count (Figure 5D). Three independent experiments were performed, and 100 cells were counted in each group for each experiment. In the control and treated groups, the mitotic index was about 5%, and there were no significant effects due to menthol treatment. The decrease in colony growth caused by menthol was not due to a decrease in cell proliferation based on Ki-67 labeling and the mitotic index data.

### Menthol-induced death in hESCs through activation of TRPM8 and TRPA1 channels

**Figure 6.**
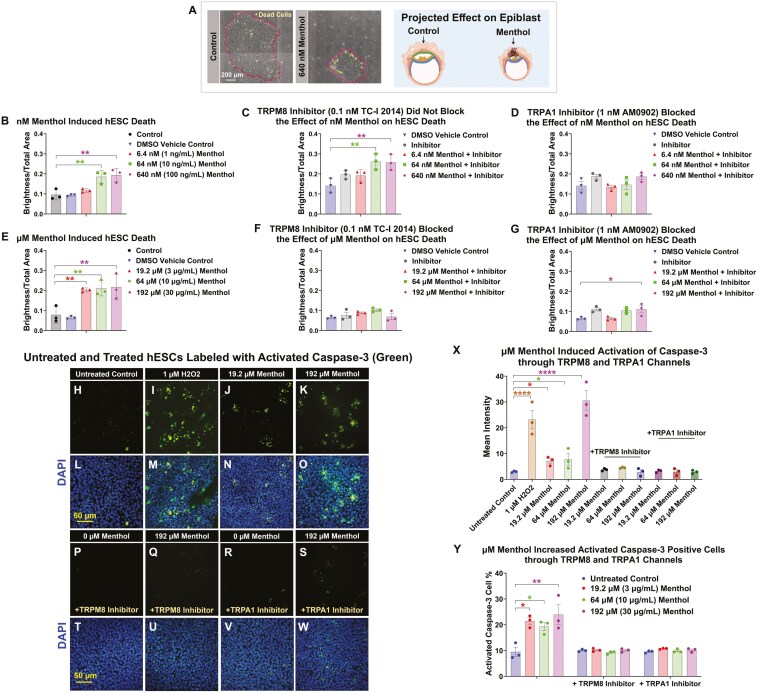
Menthol increased hESC cell death through activation of TRPM8 and TRPA1 channels. (A) Schematic representation of the cell death assay. Phase contrast micrographs showing dead cells (colorized yellow) sitting on top of hESC colonies, and diagrams showing the projected effect of cell death on the epiblast. Time-lapse data were collected at 48 or 72 hours, and cell death was quantified using the brightness-to-total area ratio feature in StemCellQC software. (B) Brightness-to-total area ratio for control, DMSO control, and nM menthol-treated groups over 72 hours. The brightness-to-total area ratio was significantly increased by nM menthol. (C) The TRPM8 inhibitor (TC-I 2014) did not block the effect of nM menthol on hESC death. (D) The TRPA1 inhibitor (AM0902) blocked the effect of nM menthol on hESC death. (E) Brightness-to-total area ratio was significantly increased in the µM menthol groups over 48 hours. (F) The TRPM8 inhibitor (TC-I 2014) blocked the effect of µM menthol on hESC death. (G) The TRPA1 inhibitor (AM0902) blocked the effect of µM menthol on hESC death. (H-K) hESC colonies were treated with 2 concentrations of menthol or H_2_O_2_ (positive control) and then labeled with an antibody to activated caspase-3 to detect apoptosis. (L-O) Images H-K merged with DAPI staining to show nuclei (blue). (P-S) hESC colonies treated with menthol in the presence of TRPM8 or TRPA1 inhibitor showing absence of caspase-3 labeling. (T-W) Images P-S merged with DAPI staining to show nuclei (blue). (X) Graph of raw data showing the intensity of activated caspase-3 labeling in the menthol treated groups ± inhibitors. (Y) Percentage of activated caspase-3 cells in groups treated with different concentrations of menthol ± inhibitors. Data are plotted as means of 3 independent experiments ± SEM for each group. Data were analyzed using a one-way ANOVA with Dunnett’s post hoc comparisons to the DMSO vehicle control. Data in (D), (F), and (X) were transformed (log(*y*)) to satisfy the assumptions of ANOVA. * = *P* < .05 and ** = *P* < .01.

hESC colonies treated with menthol for 48-72 hours were evaluated for cell death using the brightness/total area ratio in StemCellQC software.^48^ As hESCs die, they stick to the top of the colony where their phase contrast brightness increases. The brightness/total area feature measured the number of bright pixels in the colony as a ratio to the total colony area (Figure 6A). In colonies treated with 64-640 nM menthol, the brightness/total area ratio was significantly increased during 72 hours of in vitro incubation (Figure 6B). Death caused by nM menthol was not blocked by the TRPM8 inhibitor (TC-I 2014) (Figure 6C) but was significantly blocked by the TRPA1 inhibitor (AM0902) (Figure 6D). Colonies treated with µM concentrations of menthol for 48 hours had a brightness/total area ratio that was significantly higher than the untreated and DMSO controls (Figure 6E), and the number of dead cells was reduced by the TRPM8 or TRPA1 inhibitors (Figure 6F, G).

Cell death was confirmed by labeling control and treated cells with an antibody to activated caspase-3 (Figure 6H-Y). Antibody labeling was significantly elevated in the positive control (H_2_O_2_) and in each menthol-treated group. Antibody binding did not occur when incubation included inhibitors of TRPM8 and TRPA1. Mean intensity was significantly elevated in H_2_O_2_ and menthol-treated cells, but not in cells that included TRPM8 or TRPA1 inhibitor (Figure 6X). The activated caspase-3 data were further analyzed by counting the number of dead cells in each group (Figure 6Y). All µM concentrations of menthol significantly increased the number of dead cells, except for cells incubated with TRPM8 or TRPA1 inhibitor (Figure 6Y).

### Menthol caused elongation of hESC colonies through activation of TRPM8 and TRPA1 channels

**Figure 7.**
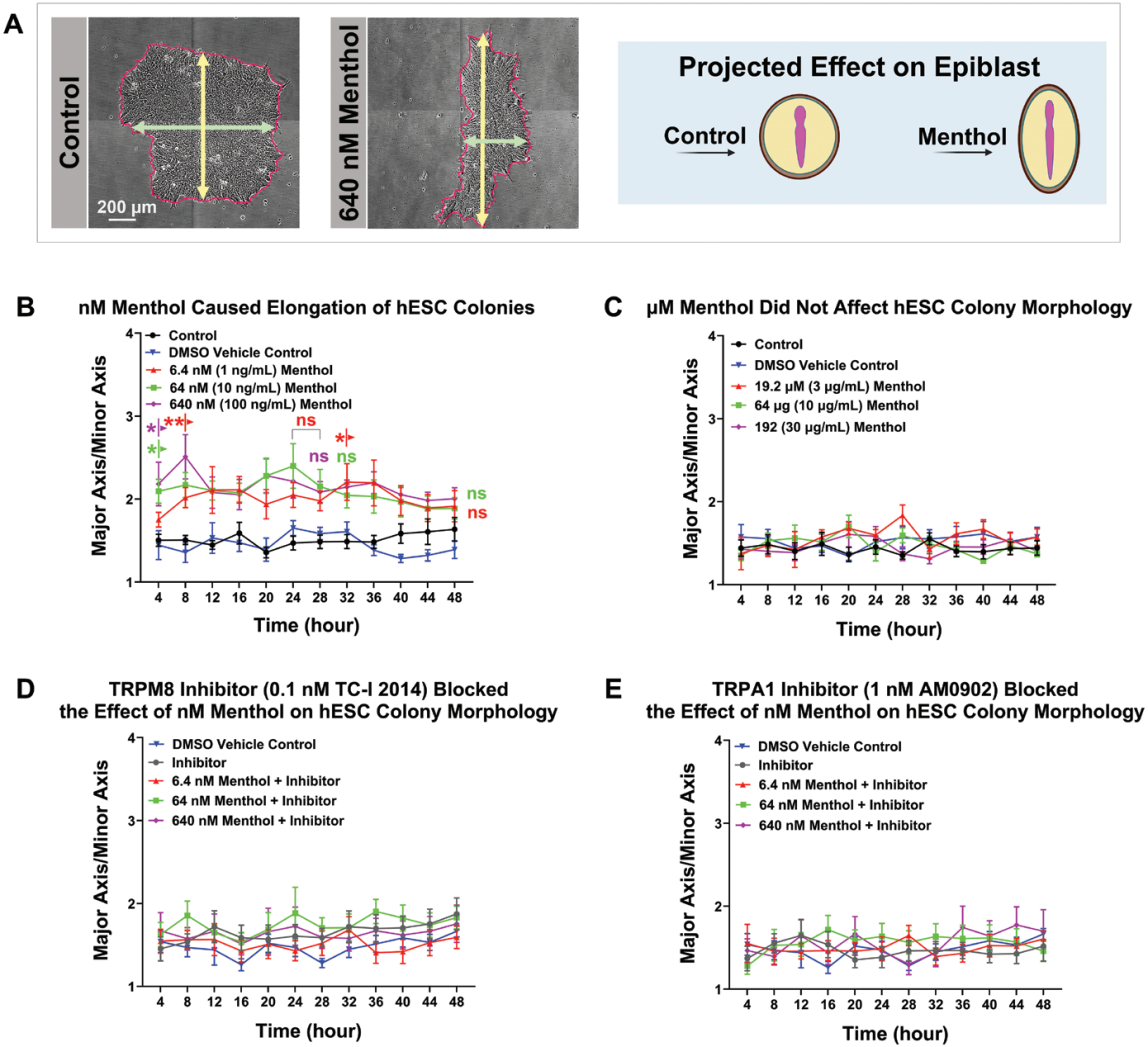
Menthol altered hESC colony morphology through activation of TRPM8 and TRPA1 channels. (A) Schematic representation of the major/minor axis assay. The major/minor axis feature was extracted using StemCellQC software. Changes in hESC colony morphology were projected for the epiblast. (B) The major/minor axis ratio over 48 hours for control and nM menthol-treated colonies. nM menthol significantly increased the ratio. (C) The major /minor axis ratio over 48 hours for control and µM menthol-treated colonies. µM menthol did not significantly affect the ratio. (D) TRPM8 inhibitor (TC-I 2014) blocked the effect of nM menthol on hESC colony morphology. (E) TRPA1 inhibitor (AM0902) blocked the effect of nM menthol on hESC colony morphology. Data are plotted as means of 3 independent experiments ± SEM for each concentration. Arrows indicate first values that differed significantly from the DMSO vehicle control by 2-way ANOVA and Dunnett’s post hoc test. Data were transformed (log(*y*)) to satisfy the assumptions of ANOVA. * = *P* < .05 and ** = *P* < .01.

Healthy undifferentiated hESC colonies are round and have tightly packed small cells with little cytoplasm.^49,54^ To determine if menthol affects hESC colony phenotype, the major/minor axis ratio was extracted from time-lapse videos using StemCellQC software (Figure 7A).^48^ nM concentrations of menthol, but not the DMSO control, significantly increased the major/minor axis ratio at most time points, indicating these colonies were elongated, rather than circular in shape (Figure 7B). In contrast, µM menthol did not significantly affect colony morphology (Figure 7C). The TRPM8 and TRPA1 channel inhibitors both independently blocked the effect of nM menthol on colony morphology (Figure 7D, E). These data show that nM menthol elongates hESC colony morphology by activating either TRPM8 or TRPA1 channels.

### Menthol increased hESC colony motility through activation of TRPM8 channels

**Figure 8.**
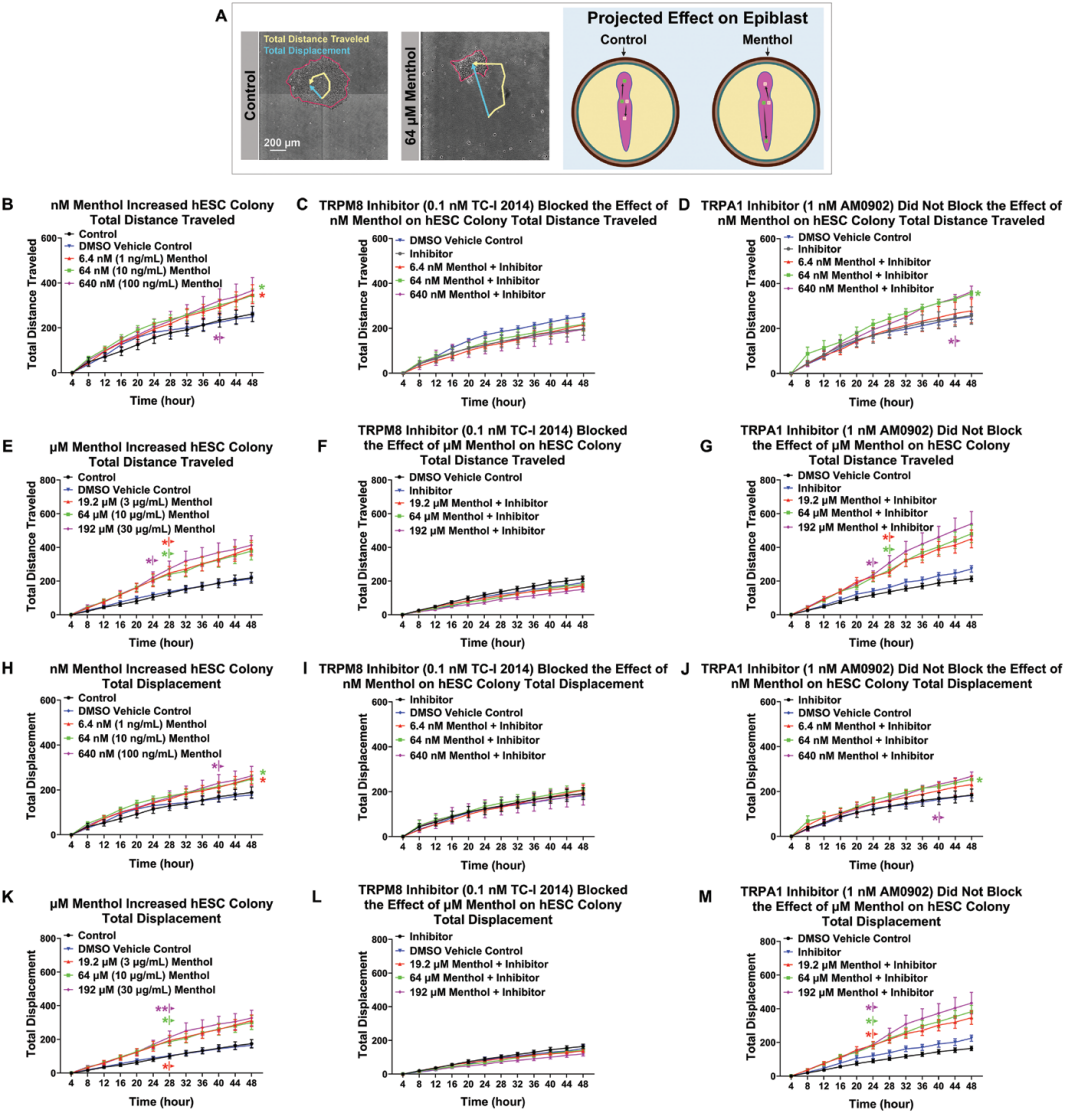
Menthol affected hESC colony motility through the activation of TRPM8 channels. (A) Schematic representation of motility feature assays. The total distance traveled and the total displacement of hESC colonies were extracted using StemCellQC software. The increase in hESC colony motility could result in the mislocation of primitive streak derivatives during gastrulation. (B) Total distance traveled over 48 hours for control and nM menthol-treated colonies. nM menthol significantly increased the total distance traveled for hESC colonies. (C) TRPM8 inhibitor (TC-I 2014) blocked the effect of nM menthol on the total distance traveled. (D) TRPA1 inhibitor (AM0902) did not block the effect of nM menthol on the total distance traveled. (E) µM menthol significantly increased the total distance traveled in hESC colonies over 48 hours. (F) TRPM8 inhibitor (TC-I 2014) blocked the effect of µM menthol on the total distance traveled. (G) TRPA1 inhibitor (AM0902) did not block the effect of µM menthol on the total distance traveled. (H) nM menthol significantly increased hESC colony total displacement over 48 hours. (I) TRPM8 inhibitor (TC-I 2014) blocked the effect of nM menthol on total displacement. (J) TRPA1 inhibitor (AM0902) did not block the effect of nM menthol on total displacement. (K) µM menthol significantly increased hESC colony total displacement over 48 hours. (L) TRPM8 inhibitor (TC-I 2014) blocked the effect of µM menthol on total displacement. (M) TRPA1 inhibitor (AM0902) did not block the effect of µM menthol on total displacement. Data are plotted as means of 3 independent experiments ± SEM. Arrows indicate first values that differed significantly from the DMSO vehicle control by 2-way ANOVA and Dunnett’s post hoc test. Data in (C), (H), and (I) were transformed (log(y)) to satisfy the assumptions of ANOVA. * = *P* < .05 and ** = *P* < .01.

“Total Distance Traveled” measures the entire trajectory of colony movement, while “Total Displacement” measures how far a colony moved from its original starting point. Alterations in these motility features could potentially affect gastrulation (Figure 8A). DMSO did not affect either motility feature when compared to the untreated control (Figure 8B). nM menthol significantly increased the total distance traveled (Figure 8B) and total displacement (Figure 8H) relative to the DMSO vehicle control. The TRPM8 inhibitor (TC-I 2014) (Figure 8C, I), but not the TRPA1 inhibitor (AM0902) (Figure 8D, J), restored both motility features to DMSO control values.

Colonies treated with µM menthol traveled a longer distance and moved further from their point of origin than untreated and DMSO control colonies (Figure 8E, K). These effects were blocked by the TRPM8 (Figure 8F, L), but not the TRPA1 (Figure 8G, M), inhibitor.

## Discussion

Menthol activated TRPA1 and TRPM8 channels in hESCs at concentrations that are likely to reach the embryo in pregnant women who vape. nM menthol induced calcium influx via TRPA1 channels, while activation of TRPM8 required µM menthol. The functions of TRPA1 and TRPM8 in human embryos are not well understood, but their changes in expression in early development and their localization in different regions of the cells, as shown in the scRNA-seq and immunocytochemistry data, indicate they play critical and varied roles. Unscheduled activation of these channels by menthol inhibited colony growth (by increasing cell death), elongated colony morphology, increased colony motility, and decreased mitochondrial reductase activity, processes critical for gastrulation. Given the high concentrations of menthol in many EC aerosols and the low concentrations that induce Ca^2+^ influx and alter cell processes, it is likely that human embryos would be adversely affected in pregnant women who vape ECs containing menthol at concentrations ≥1 mg/mL.

Our study focused on TRPA1 and TRPM8 because antagonists that block these channels inhibited menthol’s effects. Moreover, menthol activates these 2 TRPs in other cell types.^55-58^  Supplementary Table S2 shows menthol’s lowest adverse effect level (LOAEL) for TRPA1 and TRPM8 for each endpoint. All assays were affected by nM concentrations of menthol, except the MTT assay, which is generally less responsive than the other endpoints.^59^ The requirement for µM concentrations of menthol in the MTT assay may be influenced by the reliance of hESCs on glycolysis rather than mitochondrial respiration.^60^ All assays, except for the MTT and death, were affected by 6.4 or 64 nM menthol, although responses were usually stronger with µM concentrations (eg, in the growth assay). For nM concentrations, all assays, except the motility features, were affected by activation of TRPA1. The motility assays were affected by 6.4 nM menthol; however, activation occurred only via TRPM8. Since blockage of TRPA1 had no effect on motility, the pathways downstream from calcium influx likely differed for motility and the other endpoints.

TRPM8 was partially activated by nM menthol in the calcium influx assay, perhaps because the delivery of menthol was pulsatile. The morphology and motility endpoints were measured during continual incubation of cells in media containing menthol, which may allow activation of TRPM8 at nM concentrations. Delivery of menthol via culture medium resembles in vivo exposure when menthol in a mother’s blood is continually delivered to the embryo by the primitive placenta. Our exposure model estimated nM concentrations of menthol in maternal blood following inhalation of menthol-containing aerosol. However actual concentrations in the embryo/fetus may be higher. Menthol is a lipophilic molecule capable of interacting with biological membranes (*n*-octanol-water partition coefficient = 3.4),^61^ which may facilitate passage through the placenta. In humans, nicotine was detected in fetal circulation at levels exceeding maternal blood concentrations by 15%.^62,63^

Activation of TRPA1 and TRPM8 by menthol affected downstream processes that are critical in gastrulation. hESC colony growth was significantly inhibited by nM and µM menthol. Two independent assays indicated this decrease was not due to inhibition of cell proliferation. The large decrease in colony area in µM menthol was due to increased cell death, which was blocked by both the TRPA1 and TRPM8 inhibitors. Similar increases in cell death via activation of TRPM8 by menthol occurred in cancer cells.^64^ While the positive correlation between smoking tobacco cigarettes and small-for-dates babies is well documented,^65,66^ the effects of vaping on embryo/fetal growth are mixed. Our data support the conclusion that pregnant women who vape menthol-flavored ECs inhibit the growth of their embryos by increasing cell death, which may explain why some pregnant women who vaped gave birth to low-birth-weight babies.^15^ One study attributed fetal death to the use of mentholated ECs.^17^ The mixed results in the epidemiological studies may be influenced by the EC type that pregnant women used. Some ECs do not contain menthol,^59^ and these may not affect embryonic growth.

hESC colony morphology (major/minor axis ratio) was altered by activation of TRPA1 and TRPM8 channels with nM menthol. Colony elongation probably did not occur in µM treated groups because they did not grow enough to show this effect. If similar alterations in morphology occur in vivo in gastrulating embryos, the epiblast could become misshapen and interfere with normal development.

Gastrulation involves the movement of epiblast cells to form the definitive endoderm and the mesoderm.^67^ Menthol increased motility in hESC colonies through concentration-dependent activation of TRPM8 channels. Altering motility of the epiblast could impact cell migration during gastrulation, resulting in improper apportioning of cells within the trilaminar embryonic disc. Although not part of the current study, neural crest cells also undergo extensive migration,^68^ which may likewise be altered by menthol exposure. Menthol activation of TRPM8 similarly enhanced motility of squamous cell carcinoma lines by increasing matrix metallopeptidase-9 activity^69^ and knockdown or antagonism of TRPM8 inhibited migration of osteosarcoma cancer cell lines.^70^ The migration of epiblast cells is an evolutionarily conserved process, and its dysregulation could cause under or overdevelopment of organs.

Menthol is present in some EC refill fluids at concentrations as high as 84 mg/mL,^19^ which is well above 100 µg/mL (640 µM) that decreased mitochondrial reductase activity in the MTT assay. Based on our exposure model, it is unlikely that 100 µg/mL (640 µM) menthol would reach the embryo following menthol-flavored aerosol inhalation via ECs. nM concentrations of menthol did not produce a significant effect on the MTT assay; however, chronic in vivo exposure to nM menthol may be more damaging to the mitochondria in hESCs than the MTT assay reveals. Our results demonstrated that µM menthol induced mitochondrial dysfunction mainly through activation of TRPA1 and TRPM8 in hESCs. Comparable outcomes were observed in T24 cells when exposed to 1 mM menthol, which interfered with mitochondrial function through activation of TRPM8 channels.^64^

An embryo, in a pregnant woman who vapes, would likely be exposed over many days, and the effective concentrations of menthol could be lower and the responses stronger than in our study, in which exposures were at a maximum of 72 hours. EC aerosols contain other chemicals, such as benzoic acid, that could alter the effects of menthol when tested by itself.^71^ Given individual differences in vaping topography^42,72^ and the vast array of EC products,^73-75^ the effects of menthol may vary with individual pregnant women and may be affected by chronic health conditions.

## Conclusion

Exposure to nM menthol disrupted normal growth, morphology, and migration of hESC colonies by activation of TRPA1 and TRPM8 channels, which may impair successful gastrulation. Our study aligns well with a recent epidemiological study in which the OR (3.27) for fetal death was much higher when only menthol-flavored ECs were included in the analysis of birth outcomes.^17^ Both experimental and clinical data now support the conclusion that women should not vape menthol or mint flavored ECs during pregnancy.

## Supplementary Material

szae099_suppl_Supplementary_Tables_S1-S2_Texts_S1-S3_Figures_S1-S2

## Data Availability

The data supporting this study are contained within this article.
