# Supplementary material for: Menthol, a consumer product additive, adversely affects human embryonic stem cells via activation of TRPM8 and TRPA1 channels
Source: Stem Cells Transl Med. 2025 Mar 26;14(3):szae099. doi: 10.1093/stcltm/szae099 (PMC11943479; doi:10.1093/stcltm/szae099)
Supplement: szae099_suppl_Supplementary_Tables_S1-S2_Texts_S1-S3_Figures_S1-S2 [file szae099_suppl_supplementary_tables_s1-s2_texts_s1-s3_figures_s1-s2.docx]

**Supplementary Material**

**Text S1: hESC Cell Culture**

hESCs were cultured in incubators maintained at 37°C with a 95% relative humidity and 5% CO_2_ atmosphere and checked daily for normal morphology. hESCs at 80-90% confluency were passaged by a brief wash in Dulbecco’s phosphate-buffered saline without calcium or magnesium followed by a 5-minute incubation in ReLeSR to dissociate the colonies and a 1-minute incubation in Accutase to enzymatically detach the cells. Cells were pelleted by centrifugation and counted using a BioMate 3S Spectrophotometer, then seeded at 2×10^4^ cells/well in 96-well plates, 5×10^4^ cells/chamber in 8-well chamber slides, or 1×10^5^ cells/well in 12-well plates. hESC authenticity was confirmed using Short Tandem Repeat (STR) profiling (WiCell, SOP-CH-305). Routine testing of cultures ensured that cells were consistently negative for Mycoplasma contamination.

**Text S2: Immunocytochemistry Labeling and Analysis**

Primary antibodies were added in staining solution PBS-T (DPBS + 0.1% Tween) and incubated overnight at 4°C. The presence and distribution of TRP channels was studied using a rabbit polyclonal antibody to human TRPA1 (1:200), a rabbit polyclonal antibody to human TRPM1 (1:200), a rabbit polyclonal antibody to human TRPM2 (1:200), a rabbit polyclonal antibody to human TRPM3 (1:200), a rabbit polyclonal antibody to human TRPM7 (1:200), and a rabbit polyclonal antibody to human TRPM8 (1:200). Cell proliferation was evaluated using a rabbit monoclonal antibody against human Ki-67 (1:250). Cells labeled with primary antibodies were washed with PBS-T, then labeled with Alexa fluor-488 (1:500) secondary antibody for 2 hours at room temperature in staining solution. Cells were covered with Vectashield with DAPI to minimize photobleaching and to label nuclei with DAPI. Images were captured with a Nikon Eclipse Ti inverted microscope equipped with a high-resolution Andor Zyla VSC-04941 camera. Images were processed in Nikon NIS-Elements AR.

**Text S3: Mitochondrial Reductase Activity Assayed Using the MTT Assay**

Tetrazolium salt methyl thiazoyl tetrazolium (MTT) measured mitochondrial reductase activity in hESCs by the reduction of tetrazolium dye MTT to a purple color formazan. hESCs (2 × 10^4^ cells/well) in 96-well plates were allowed to attach for 48 hours and then exposed to nM and µM menthol dissolved in dimethyl sulfoxide (DMSO). The 96-well plates were laid out to have negative controls (culture medium only, and DMSO vehicle control) in columns 1 and 2, followed by serial dilutions of menthol. After 24 hours of exposure, 20 µL of MTT dissolved in 5 mg/mL of DPBS were added to each well and the plates were incubated for 2 hours at 37°C. Solutions were then removed and 100 µL of DMSO were added to each well and plates were gently rocked for 15 minutes until homogenous. Absorbance was read at 570 nm with a BioTek Synergy HTX Microplate Reader. In experiments involving TRP channels inhibitors, concentration-response curves were obtained using the MTT assay in the presence of serial dilutions of TRPA1 inhibitor (AM 0902), TRPM2 inhibitor (tatM2NX), TRPM3 inhibitor (Mefenamic Acid), TRPM7 inhibitor (CCT128930), TRPM8 inhibitor (TC-I 2014), and a cocktail of both TRPA1 and TRPM8 inhibitors. hESCs were pre-incubated with inhibitor(s) for 20 minutes prior to their exposure to menthol at a concentration equal to its IC_50_ value determined by the MTT assay. Concentration-response curves were generated using Graphpad Prism.

**Table S1. Key Resources Table**

| Reagent Resource | Source | Identifier |
| --- | --- | --- |
| Antibodies | | |
| TRPA1 Antibody | Santa-Cruz Biotechnology, Heidelberg, Germany | cat. no. sc-376495 |
| TRPM1 Antibody | Biorbyt, Cambridge, UK | cat. no. orb422930 |
| TRPM2 Antibody | Biorbyt, Cambridge, UK | cat. no. orb373346 |
| TRPM3 Antibody | Alomone Lab, Jerusalem, Israel | cat. no. ACC-050 |
| TRPM7 Antibody | Alomone Lab, Jerusalem, Israel | cat. no. ACC-047 |
| TRPM8 Antibody | Abcam, Cambridge, MA, USA | cat. no. ab109308 |
| Ki-67 Antibody | Abcam, Boston, MA, USA | cat. No. ab16667 |
| Alexa Fluor-488 Antibody | Thermo Fisher Scientific, Houston, TX, USA | cat. no. A21206 |
| TRPA1 (Extracellular) Antibody | Alomone Lab, Jerusalem, Israel | cat. no. ACC-037 |
| TRPM8 (Extracellular) Antibody | Alomone Lab, Jerusalem, Israel | cat. no. ACC-049 |
| Rabbit IgG Isotype Control | Thermo Fisher Scientific, Chino, CA, USA | cat. no. 02-6102 |
| Activated Caspase-3 (Asp175) | Cell Signaling Technology, Danvers, MA, USA | cat. no. 9661 |
| Experimental Models: Cell Lines | | |
| WiCell (H9; Female) | Madison, WI, USA | cat. no. WA09 |
| Culturing Reagents and Chemicals | | |
| Dulbecco’s Phosphate-Buffered Saline Without Calcium or Magnesium | DPBS-; Lonza, Walkersville, MD, USA | cat. no. 14190144 |
| mTeSR Plus | STEMCELL Technologies, Vancouver, BC, Canada | cat. no. 100-0276 |
| Matrigel | Corning, New York, NY, USA | cat. no. 354234 |
| ReLeSR | STEMCELL Technologies, Vancouver, BC, Canada | cat. no. 05872 |
| Accutase | Innovative Cell Technologies, San Diego, CA, USA | cat. no. AT-104 |
| Donkey Serum | Sigma-Aldrich, St. Louis, MO, USA | cat. no. D9663 |
| Vectashield with DAPI | Vectashield, San Francisco, CA, USA | cat. no. H-1200 |
| Menthol (L(-)-Menthol) | Thermo Fisher Scientific, Chino, CA, USA | cat. no. AC125401000 |
| MTT (Methyl Thiazoyl Tetrazolium) | Sigma-Aldrich, St. Louis, MO, USA | cat. no. M5655 |
| DMSO (Dimethyl Sulfoxide) | ATCC, Manassas, VA, USA | cat. no.4-X |
| TRPA1 Inhibitor (AM 0902) | Tocris Bioscience, Minneapolis, MN, USA | cat. no. 59-141-0 |
| TRPM2 Inhibitor (tatM2NX) | Isca Biochemicals, Plymouth, PA, USA | cat. no. TP-010 |
| TRPM3 Inhibitor (Mefenamic Acid) | Thermo Fisher Scientific, Chino, CA, USA | cat. no. AAJ6270514 |
| TRPM7 Inhibitor (CCT128930) | Abcam, Cambridge, MA, USA | cat. No. ab273533 |
| TRPM8 Inhibitor (TC-I 2014) | Tocris Bioscience, Minneapolis, MN, USA | cat. no. 5410 |
| HHBS (Hanks' Buffer with 20 mM HEPES C_8_H_18_N_2_O_4_S) | AAT Bioquest, Sunnyvale, CA, USA | cat. no. 20011 |
| H_2_O_2_ | Rite Aid, Camp Hill, PA, USA | cat. no. NDC 11822-3155-3 |
| Equipment | | |
| BioMate 3S Spectrophotometer | Thermo Fisher Scientific, Chino, CA, USA |  |
| Nikon Eclipse Ti Inverted Microscope | Nikon Instrument, Melville, NY, USA |  |
| Andor Zyla VSC-04941 Camera | Andor, Belfast, UK |  |
| Synergy HTX Microplate Reader | BioTek, Winooski, VT, USA |  |
| Nikon BioStation CT | Nikon Instruments, Melville, NY, USA |  |
| Consumables | | |
| 8-well Chamber Slides | Ibidi, Gräfelfng, Germany | cat. no. 80826-90 |
| 96-well Clear Bottom Black Plates | Greiner Bio-One, Monroe, NC, USA | cat. no. 655090 |
| Software and Algorithms | | |
| Nikon NIS-Elements AR | Nikon | v.4.60.00 |
| MATLAB R2022b | MATLAB | v.9.13 |
| MATLAB Runtime R2022b | MATLAB | v.9. |
| Prism 10 | Graphpad | v.3.10.10 |
| StemCellQC | Talbot Lab | v.2-17  https://talbotlab.ucr.edu/stemcellqctm |
| ImageJ | NIH | v.1.54f |
| CL Quant | Nikon | v.3.10 |
| Critical Commercial Assays | | |
| Fluo-8 No Wash Calcium Assay Kit | Abcam, Cambridge, MA, USA | cat. no. ab112129 |

**Figure S1: Estimation of Menthol Concentration in Maternal Blood during Week 2 of Pregnancy**


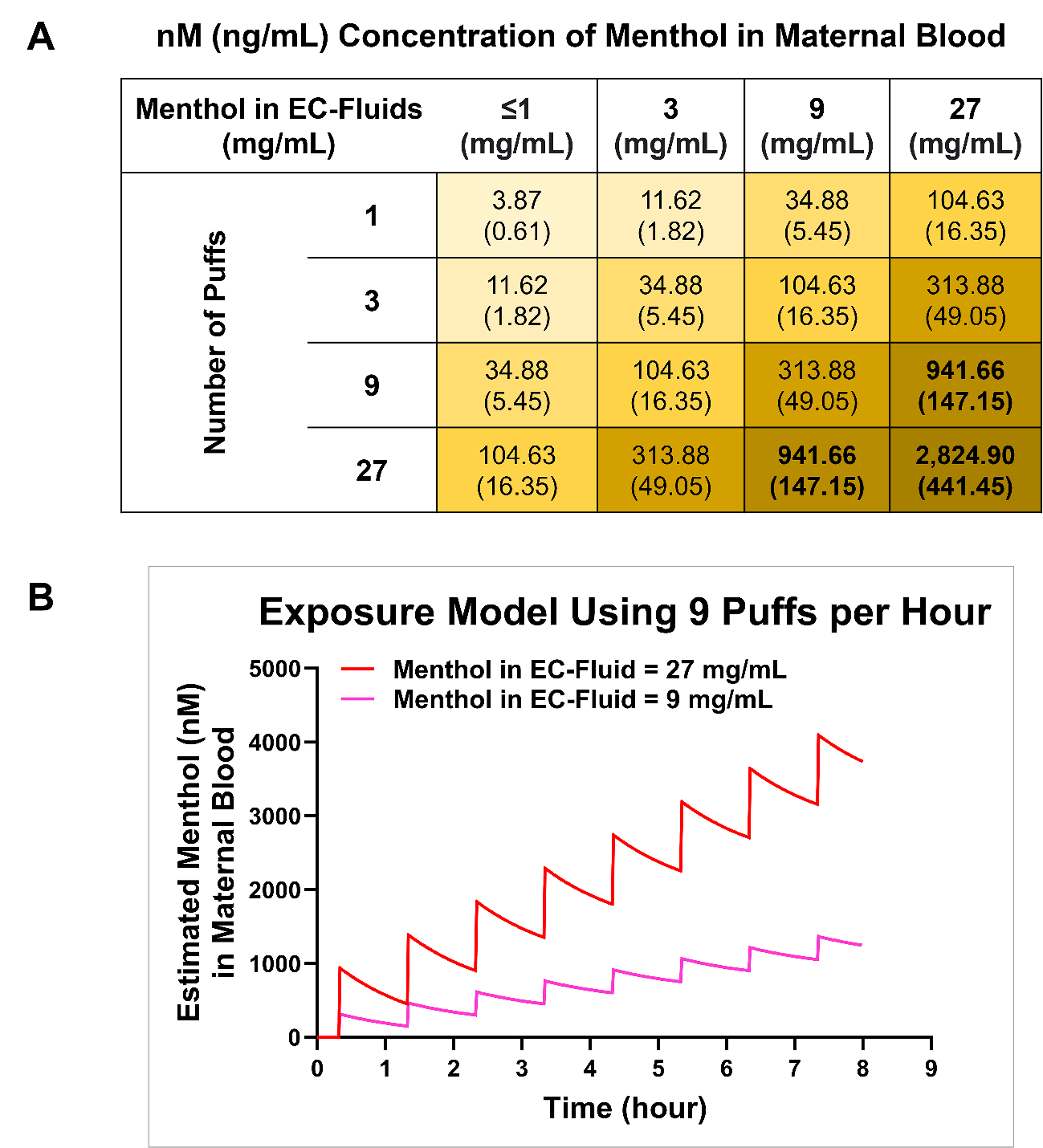


**Figure S1. Concentrations of Menthol in Maternal Blood During Week 2 of Pregnancy.** (A) Plasma menthol concentrations were estimated in the blood of pregnant women who vape ECs containing menthol. Estimates were based on different concentrations of menthol in EC-fluids and different numbers of puffs. nM and µM concentrations of menthol are likely to reach 2-week-old embryos. (B) Estimated plasma concentrations for two EC-fluids vaped at 9 puffs/hour with an estimated half-life for menthol of about 50 minutes.

**Figure S2: Menthol-induced Calcium Influx in hESCs was Blocked by Antibodies to TRPM8 and TRPA1 Channels.**


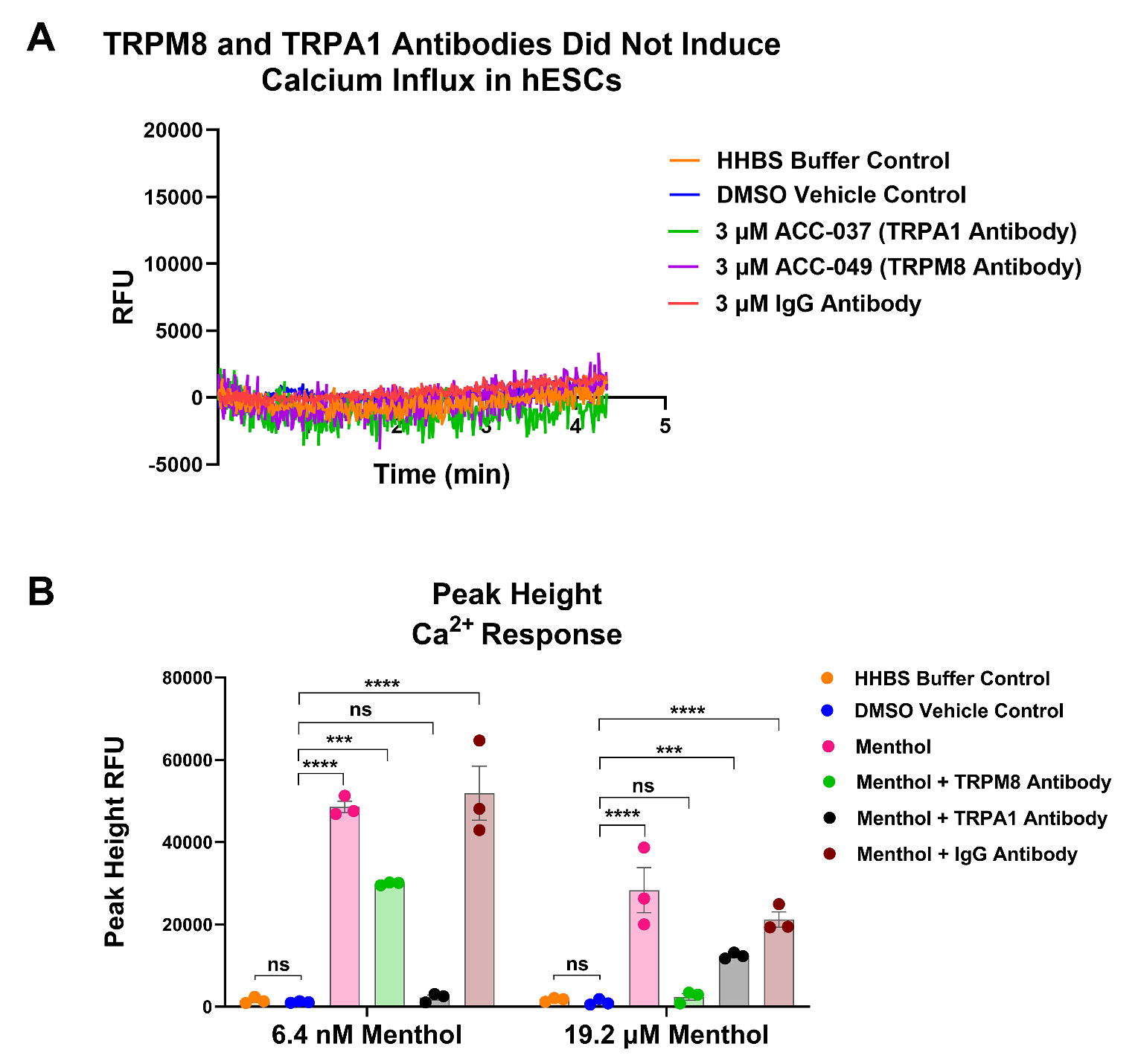


**Figure S2. Antibodies Inhibited Menthol Activation of TRPM8 and TRPA1 Channels.** (A) DMSO vehicle control and 3 µM of the TRPM8 antibody (ACC-049), TRPA1 antibody (ACC-037), and control IgG antibody did not induce an increase in intracellular calcium in hESCs. (B) Mean peak heights for hESCs treated with 6.4 nM or 19.2 µM menthol ± TRP antibodies. TRPA1 antibody (ACC-037) significantly blocked calcium influx (~95% reduction in peak height) induced by 6.4 nM menthol, while TRPM8 antibody (ACC-049) significantly blocked calcium influx (~92% reduction in peak height) at a higher menthol concentration of 19.2 µM. Control IgG antibody did not block activation of any of the channels by menthol. A one-way ANOVA was performed on transformed data (log(y)) with Dunnett’s posthoc comparisons to the DMSO vehicle control. ***p<0.001, and ****p<0.0001. n.s. = not significant.

**Table S2: Hierarchy of Potency for Endpoint Assays Based on LOAELs^1^**

| **Assay** | **nM Concentrations** | **µM Concentrations** |
| --- | --- | --- |
| **Calcium Influx Assay** | 6.4 nM via TRPA1 | 19.2 µM via TRPM8 +TRPA1 |
| **Growth Inhibition** | 6.4 nM via TRPA1 | 19.2 µM via TRPM8 + TRPA1 |
| **Altered Morphology** | 6.4 nM via TRPA1 or TRPM8 | - |
| **Total Distance Traveled** | 6.4 nM via TRPM8 | 19.2 µM via TRPM8 |
| **Total Displacement** | 6.4 nM via TRPM8 | 19.2 µM via TRPM8 |
| **Death** | 64 nM via TRPA1 | 19.2 µM via TRPM8 or TRPA1 |
| **MTT** | nM No Effect | 640 µM via TRPM8 + TRPA1 |

^1^ LOAEL refers to the lowest adverse effect level.
